# Transfer of maternal immunity using a polyvalent vaccine and offspring protection in Nile tilapia, *Oreochromis niloticus*

**DOI:** 10.12688/f1000research.52932.4

**Published:** 2023-11-10

**Authors:** Amrullah Amrullah, Wahidah Wahidah, Ardiansyah Ardiansyah, Indrayani Indrayani

**Affiliations:** 1 Aquaculture, Pangkep State Polytechnic of Agriculture, Pangkep, South Sulawesi, 90655, Indonesia; 2 Agricultural Technology Education, Makassar State University, Makassar, South Sulawesi, Indonesia

**Keywords:** Aeromonas hydrophila, bivalent vaccine, monovalent vaccine, Pseudomonas fluorescens, Streptococcus agalactiae.

## Abstract

**Background:**

Vaccination is an effective and alternative means of disease prevention, however, it cannot be conducted on the offspring of fish. For this process to take place, the transfer of maternal immunity should be implemented. This study aims to determine the effectiveness of transferring immunity from the broodstock to the offspring using a polyvalent vaccine against *Aeromonas hydrophila, Streptococcus agalactiae,* and *Pseudomonas fluorescens* in Nile tilapia, *Oreochromis niloticus.*

**Methods:**

Nile tilapia broodstock with an average weight of 203g (±SD 23) was reared in spawning ponds until mass spawning and harvested one week post-spawning for vaccination. After being vaccinated according to the treatment, each fish broodstock was reared in 3x3 m cages installed in an earthen pond with a density of 20 broodstock, consisting of 15 females and 5 males. The vaccine used was a formalin-killed whole-cell vaccine at a density of 10 ^10^ cfu/mL injected intramuscularly ( *i.m.*) at a dose of 0.4 mL/kg fish. Nile tilapia was injected with a vaccine used as a treatment. Example include *A. hydrophila* monovalent (MA) *, S. agalactiae* monovalent (MS) *, P. fluorescens* monovalent (MP), *A. hydrophila* and *S. agalactiae* bivalent (BAS) *, A. hydrophila* and *P. fluorescens* bivalent (BAP), *P. fluorescens* and *S. agalactiae* bivalent (BPS), and *A. hydrophila, S. agalactiae,* and *P. fluorescens* polyvalent vaccines (PAPS). While the control was fish that were injected with a PBS solution. The broodstock’s immune response was observed on the 7 ^th^, 14 ^th^, 21 ^st^, and 28 ^th^ days, while the immune response and challenge test on the offspring was conducted on the 10 ^th^, 20 ^th^, 30 ^th^, and 40 ^th^ day during the post-hatching period. The parameters observed consisted of total leukocytes, phagocytic activity, antibody titer, lysozyme, and relative survival percentage (RPS).

**Result:**

The application of PAPS in broodstock could significantly induce the best immune response and immunity to multiple diseases compared to other treatments. The RPS of the PAPS was also higher than the other types of vaccines. This showed that the transfer of immunity from the broodstock to the Nile tilapia offspring could protect it against bacterial diseases such as *A. hydrophila*, *S. agalactiae*, and *P. fluorescens*.

**Conclusion:**

The application of polyvalent vaccine *A. hydrophila, S. agalactiae, P. fluorescens* vaccines increased the broodstock’s immune response and it was transferred to their offsprings. Polyvalent vaccines derived from maternal immunity can protect offspring from disease up to 30 days of age. They were able to produce tilapia seeds that are immune to diseases caused by *A. hydrophila, S. agalactiae*, and *P. fluorescens.*

## Introduction

Tilapia was originally considered to be more resistant to bacterial, parasitic, fungus, and viral diseases than other species of cultivated fish. However, they are found to be susceptible to bacterial and parasitic diseases ^1– 3^, particularly during the offspring phase ^4^. Globally, the control of bacterial disease mostly uses antibiotics that are proven not environmentally friendly ^5– 7^. Some common diseases of tilapia found in several Southeast Asian countries including Indonesia are *Streptococcus agalactiae*, *Aeromonas hydrophila*, *Edwardsiella ictaluri*, *Flavobacterium columnaris*, and *Pseudomonas fluorescens* infections ^8– 10^. In addition to the bacterial disease, a new disease has emerged called Tilapia Lake Virus (TiLV) disease whose specific host is tilapia, causing disease outbreaks with high mortality rates in several Southeast Asian countries such as Thailand ^11^ and Malaysia ^12^.

Among the various methods of disease control, vaccination is one of the most effective ways, which is commonly used ^5, 13– 16^. The administration of vaccines is meant to produce antibodies that could improve the immunity of tilapia ^3, 5^. Unfortunately, they could not be administered to the offspring of fish because the organs that form the immune response are not yet fully developed, therefore they are unable to produce antibodies ^7, 13– 17^. Tilapia fry was not able to produce their own immune system at the age of less than 21 days ^18^, Immune systems of *Xenopus laevis* develop within 2 weeks of age ^19^, while Indian major carp develop within 3 weeks of age ^20^.

An effective solution to the aforementioned issue is the application of maternal immunity transfer. This is the transfer of immunity from broodstock to offspring, by which immunoglobulin (IgM type) are transferred through eggs ^19, 21, 22^. Maternal immunity has been shown to improve the fish offspring’s immunity against pathogens in the early phases of their lives ^23– 26^.

This process is usually carried out using monovalent vaccines ^27– 30^. However, a polyvalent vaccine would be more effective because it could control multiple diseases ^3, 31, 32^ especially using a formalin-killed vaccine with low production cost compared to other types of vaccines ^3^. Though the effectiveness has been known, the application of polyvalent vaccines to confer maternal immunity in offspring has not been extensively investigated, particularly in Nile tilapia ( *O. niloticus*).

The transfer of maternal immunity using polyvalent vaccine for *S. agalactiae*, *Lactococcus garvieae*, and *Enterococcus faecalis* has been studied by Abu-elala *et al.* ^33^, and three vaccine strains for *S. agalactiae* by Nurani *et al.* ^34^. The types of bacterial diseases studied in the aforementioned studies are very limited even though Nile tilapia often suffer from them in fish farms and hatcheries ^35^. Besides being infected by *S. agalactiae* ^29, 34– 36^, Nile tilapia are often infected by *A. hydrophila* ^9, 35, 37^ and *P. fluorescens* ^37, 38^ leading to high mortality, including in Indonesia. Therefore, this study aimed to examine maternal immunity transfer using the polyvalent vaccine for *S. agalactiae*, *A. hydrophila,* and *P. fluorescens* (PAPS). It was expected that the broodstock could pass their immunity to their offspring, making them resistant to the three diseases ( *A. hydrophila, S. agalactiae*, and *P. fluorescens* bacteria), and also the production of tilapia offspring could also be increased. Furthermore, this study aimed to determine the effectiveness of the transfer of immunity induced by PAPS against *A. hydrophila, S. agalactiae,* and *P. fluorescens* from the Nile tilapia ( *O. niloticus*) broodstock to their offspring and the protection against *S. agalactiae, A. hydrophila,* and *P. fluorescens* infections.

## Methods

### Experimental animal

Nile tilapia broodstock, obtained from the Ompo Inland Hatchery, Soppeng, Indonesia, with an average weight of 203g (±SD 23) were used as experimental animal. They were kept in spawning ponds (25X30X1.2 LxWxD) and fed *ad libitum* with pellets that have a protein content of 30% in the mornings and afternoons. Also, 25% of the water was replaced daily. One week after the fish spawned, they were harvested and a large number of Nile tilapia broodstock at gonad developmental stage 2 were obtained.

### Vaccine production

Pure isolates of the *A. hydrophila*, *S. agalactiae,* and *P. fluorescens* bacteria were obtained from the Research and Development of Fish Disease Control Installation, Ministry of Marine Affairs and Fisheries, Depok, Indonesia. Vaccine production was carried out by harvesting bacteria aged 24 hours, which were cultured on TSA media. The yields were then put into 100 mL of PBS with a bacterial density of 10 ^10^ cfu/mL measured by the McFarland method. Further, it was killed with formalin according to the results of Amrullah *et al.* ^39^, *S. agalactiae* and *P. fluorescens* were inactivated in 1% formalin, while *A. hydrophila* was inactivated with 0.6% ^39^. Later, stirred and incubated for 24 hours at 4°C. After 24 hours of incubation, a vaccine safety test was carried out using the sterilization method. Finally, the vaccine was diluted at a dose of 10 ^7^ cfu/mL and was ready to be used for the vaccination of tilapia broodstock.

### Vaccine treatments and administration

The vaccine treatments consist of (1) a monovalent vaccine against *A. hydrophila* (MA), (2) a monovalent vaccine against *P. fluorescens* (MP), (3) a monovalent vaccine against *S. agalactiae* (MS), (4) a bivalent vaccine against *A. hydrophila, P. fluorescens* and (BAP), (5) a bivalent vaccine against *A. hydrophila* and *S. agalactiae* (BAS), (6) a bivalent vaccine against *P. fluorescens* and *S. agalactiae* (BPS), (7) a polyvalent vaccine against *A. hydrophila, P. fluorescens* and *S. agalactiae* (PAPS), and (8) the control, fish injected with PBS solution. However, only the female broodstock was vaccinated.

The vaccination method used was intramuscular ( *i.m.*) ^40, 41^ by injecting between the first and second scales of the dorsal fin and was administered at a dose of 0.4 mL/kg of fish (±0.08 mL/fish). After the fish were vaccinated, a booster with the same dose as the initial vaccination was later administered on the 7 ^th^ day. The fish were anesthetized using MS-222 (Sigma) before injection.

The gonad developmental stage 2 fish post-vaccination were reared using 3×3 m cages and installed in dirt ponds 25×30×1.2 (L×W×D). Furthermore, 20 broodstock were reared per cage, consisting of 15 females and 5 males. The fish were fed with pellets at a dose of 4%/day in the morning, at midday, and in the afternoon. The water was replaced daily at a rate of 5%/day. The fish would spawn after being reared for approximately 4 weeks.

### Broodstock and larvae immune response

Following vaccinations, the fish’s immune response was observed on the 7 ^th^, 14 ^th^, 21 ^st^, and 28 ^th^ day by collecting caudal vein blood samples. The immune response parameters were the antibody titer using the direct agglutination method ^42^, total leukocyte ^9, 34, 43^, phagocytic ^44, 45^ and lysozyme activities ^27, 34, 45, 46^.

Random blood sampling from the offspring was conducted on each treatment group on the 10 ^th^, 20 ^th^, 30 ^th^, and 40 ^th^ day post-hatching period. Serum was collected by grinding 5 offspring in effendorf tube for 5 µL with PBS-tween at a ratio of 4:1. It was then centrifuged at 6000 rpm. Furthermore, the serum in the second layer of the centrifugation result was harvested and stored at 47°C for 30 minutes to inactivate the complements ^47^. It was then stored for agglutination titer and lysozyme activity. The direct agglutination test on both broodstocks and offspring was carried out by adding 25 µL PBS into the microplate from the 1 ^st^ to 12 ^th^ wells. A total of 25 µL of test fish serum based on treatment was added to the 1st well (positive control) and 2 ^nd^ well. Furthermore, multilevel dilutions were carried out starting from the 2 ^nd^ well to the 11th well, while the 12 ^th^ well was not added with serum (negative control). Furthermore, 25 µL of whole cell antigen of bacteria *A. hydrophila, S. agalactiae, P. fluorescens* was added to each of the 1 ^st^ to 12 ^th^ wells ^48^. The microtiter containing antibodies and antigens was then incubated overnight at room temperature and the agglutinating titer was calculated.

### Challenge procedures

The offspring challenge test was conducted on the 10, 20, 30, and 40 days after hatching. It was carried out by dividing the fish into 7 groups based on the type of vaccine administered plus one unvaccinated. Challenge tests on all treatments were carried out using three types of pathogenic bacteria; *A. hydrophila, S. agalactiae,* and *P. fluorescens*. This test was carried out by placing 20 offsprings into containers containing 4 liters of water and then they were immersed for 24 h in water containing pathogenic bacteria at a dose of 2.1×10 ^8^ cfu/mL according to their relative treatments, each conducted triplicate. To observe the effectiveness of the vaccine, the relative percentage survival (RPS) was calculated ^49, 50^ on the 14 ^th^ day post-challenge test.

### Data analysis

The data for the specific and non-specific immune response and RPS were analyzed statistically and with Duncan’s test (IBM SPSS Statistic 21; Chicago, IL, USA).

## Results

### Broodstock total leukocyte dan phagocytic activity post-vaccination

**Figure 1.**
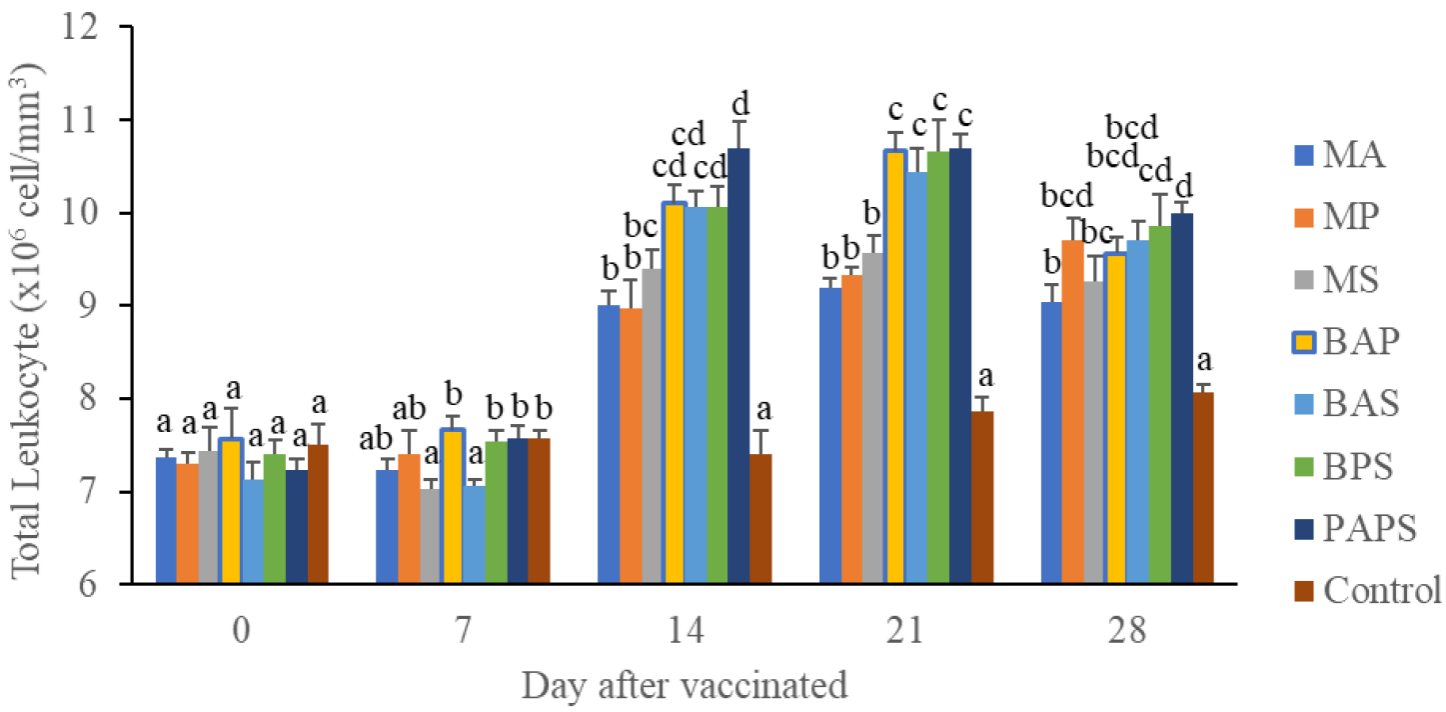
Total leukocyte of tilapia broodstock after the vaccination with various types of vaccines (mean±SE). MA: *A. hydrophila* monovalent, MS: *S. agalactiae* monovalent, MP: *P. fluorescens* monovalent, BAS: *A. hydrophila* and *S. agalactiae* bivalent, BAP: *A. hydrophila* and *P. fluorescens* bivalent, BPS: *P. fluorescens* and *S. agalactiae* bivalent, and PAPS: *A. hydrophila, S. agalactiae,* and *P. fluorescens* polyvalent vaccines. Values with different superscripts a,b indicate that their corresponding means are significantly different (P<0.05) according to one-way ANOVA followed by Duncan’s test.

**Figure 2.**
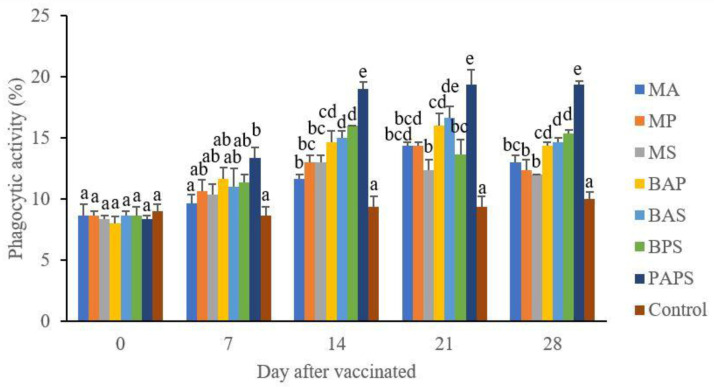
The phagocytic activity in the tilapia broodstock after being vaccinated with the various types of vaccines (mean±SE). MA: *A. hydrophila* monovalent, MS: *S. agalactiae* monovalent, MP: *P. fluorescens* monovalent, BAS: *A. hydrophila* and *S. agalactiae* bivalent, BAP: *A. hydrophila* and *P. fluorescens* bivalent, BPS: *P. fluorescens* and *S. agalactiae* bivalent, and PAPS: *A. hydrophila, S. agalactiae,* and *P. fluorescens* polyvalent vaccines. Values with different superscripts a,b indicate that their corresponding means are significantly different (P<0.05) according to one-way ANOVA followed by Duncan’s test.

In general, the different types of vaccines at each period of post-vaccination had a significant effect (P<0.05) on the broodstock's total leukocyte ( Figure 1), and phagocytic activity ( Figure 2). The follow-up test showed that the fish vaccinated with PAPS had the highest total leukocyte (7.56–10.70×10 ^6^ cell/mm ^3^) and phagocytic activity (8.33–19.33%), followed by those vaccinated with bivalent and monovalent vaccines, while the lowest was found in control (total leukocyte was 7.40–7.86×10 ^6^ cell/mm ^3^, phagocytic activity was 9.00–9.33%).

### Broodstock and offspring agglutination titers

The broodstock’s antibody ( Table 1) increased, especially after the booster, except in the unvaccinated fish. After the peak, the broodstock’s immune response remained high up to day 28 even though there was a tendency for it to decrease. All the types of vaccines at each point of time had a significant effect (P<0.05) on the agglutination titer in the broodstock. The Duncan’s follow-up test showed that the vaccinated broodstock had a higher agglutination titer than the unvaccinated fish. Also, the highest significant value was found in the vaccinated fish with PAPS (1.67–6.67), followed by those vaccinated with the bivalent and monovalent vaccines, while the lowest was in the control (1.33–1.67). Offspring from unvaccinated broodstocks have native immunity, hence in the agglutination test occurs agglutination, but it is very low and does not show an increase, and has not been able to control infections.

**Table 1.** The agglutination titer in Nile tilapia broodstock after being vaccinated with various types of vaccines (mean±SE). MA: *A. hydrophila* monovalent, MS: *S. agalactiae* monovalent, MP: *P. fluorescens* monovalent, BAS: *A. hydrophila* and *S. agalactiae* bivalent, BAP: *A. hydrophila* and *P. fluorescens* bivalent, BPS: *P. fluorescens* and *S. agalactiae* bivalent, and PAPS: *A. hydrophila, S. agalactiae,* and *P. fluorescens* polyvalent vaccines. Values with different superscripts a,b indicate that their corresponding means are significantly different (P<0.05) according to one-way ANOVA followed by Duncan’s test.

Type of vaccine	Agglutination titer (log2)				
	Day after vaccinated (day)				
	0	7	14	21	28
MA	1.67±0.33 ^a^	2.00±0.00 ^a^	3.33±0.33 ^a^	3.67±0.3 ^bc^	3.67±0.33 ^bc^
MP	1.67±0.33 ^a^	2.67±0.33 ^a^	3.67±0.33 ^a^	3.33±0.33 ^bc^	3.33±0.33 ^b^
MS	1.33±0.33 ^a^	2.33±0.33 ^a^	3.33±0.33 ^a^	3.00±0.00 ^b^	3.33±0.33 ^b^
BAP	2.00±0.58 ^a^	2.33±0.33 ^a^	4.33±0.33 ^a b^	4.33±0.33 ^c^	4.67±0.33 ^bc^
BAS	1.67±0.33 ^a^	2.33±0.33 ^a^	4.33±0.33 ^a b^	4.33±0.33 ^c^	4.33±0.88 ^bc^
BPS	1.67±0.67 ^a^	2.33±0.33 ^a^	4.33±0.33 ^a b^	4.33±0.33 ^c^	5.00±0.58 ^c^
PAPS	1.67±0.33 ^a^	3.67±0.33 ^b^	5.33±0.33 ^b^	6.67±0.33 ^d^	6.67±0.33 ^d^
Control	1.67±0.33 ^a^	1.67±0.33 ^a^	1.33±0.33 ^a^	1.33±0.33 ^a^	1.67±0.33 ^a^

Based on the effect of the vaccine on the broodstock’s immune response, the agglutination titer in the offspring from the vaccinated broodstock at ages 10, 20, 30, and 40 days was higher than unvaccinated (P<0.05). The follow-up test showed that PAPS was more effective in increasing the agglutination titer in the offspring (6.33–3.00) than the bivalent and monovalent vaccines. The results showed that the administration of vaccines in tilapia broodstock had a significant effect on the maternal immunity transfer to the offsprings that were up to 30 days old ( Table 2).

**Table 2.** The agglutination titer of tilapia offspring from maternal immunity produced by various types of vaccines at the ages of 10, 20, 30 and 40 days post-hatching (mean±SE). MA: *A. hydrophila* monovalent, MS: *S. agalactiae* monovalent, MP: *P. fluorescens* monovalent, BAS: *A. hydrophila* and *S. agalactiae* bivalent, BAP: *A. hydrophila* and *P. fluorescens* bivalent, BPS: *P. fluorescens* and *S. agalactiae* bivalent, and PAPS: *A. hydrophila, S. agalactiae,* and *P. fluorescens* polyvalent vaccines. Values with different superscripts a,b indicate that their corresponding means are significantly different (P<0.05) according to one-way ANOVA followed by Duncan’s test.

Type of vaccine	Agglutination titer (log2)			
	Day post-hatching (day)			
	10	20	30	40
MA	4.00±0.58 ^a b^	3.67±0.33 ^bc^	1.67±0.33 ^a^	1.33±0.33 ^a^
MP	4.00±0.00 ^a b^	3.67±0.33 ^bc^	1.67±0.33 ^a^	1.33±0.33 ^a^
MS	3.67±0.33 ^b^	3.33±0.33 ^b^	2.33±0.33 ^a b^	1.33±0.33 ^a^
BAP	4.67±0.33 ^a b^	4.67±0.33 ^c^	2.33±0.33 ^a b^	1.67±0.33 ^a^
BAS	5.00±0.58 ^c^	4.33±0.33 ^bc^	2.33±0.33 ^a b^	1.67±0.33 ^a^
BPS	4.33±0.33 ^a b^	4.33±0.33 ^bc^	2.33±0.33 ^a b^	1.33±0.33 ^a^
PAPS	6.33±0.33 ^d^	5.67±0.33 ^d^	3.00±0.33 ^b^	1.67±0.33 ^a^
Control	1.67±0.33 ^a^	1.67±0.33 ^a^	1.67±0.33 ^a^	1.33±0.33 ^a^

### Broodstock and offspring lysozyme activity

**Figure 3.**
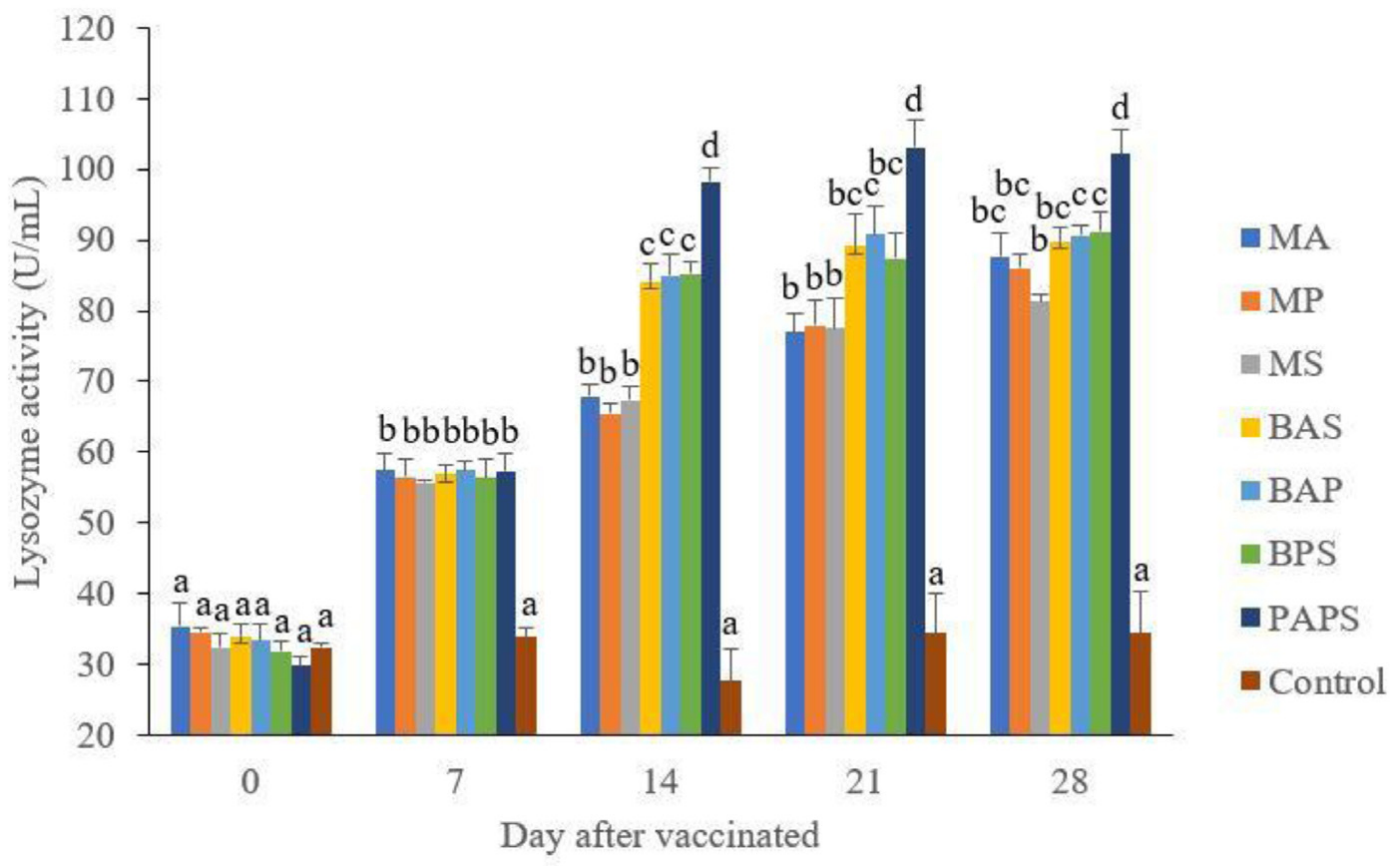
The lysozyme activity in the tilapia broodstock after being vaccinated with the various types of vaccines (mean±SE). MA: *A. hydrophila* monovalent, MS: *S. agalactiae* monovalent, MP: *P. fluorescens* monovalent, BAS: *A. hydrophila* and *S. agalactiae* bivalent, BAP: *A. hydrophila* and *P. fluorescens* bivalent, BPS: *P. fluorescens* and *S. agalactiae* bivalent, and PAPS: *A. hydrophila*, *S. agalactiae*, and *P. fluorescens* polyvalent vaccines. Values with different superscripts a,b indicate that their corresponding means are significantly different (P<0.05) according to one-way ANOVA followed by Duncan’s test.

**Figure 4.**
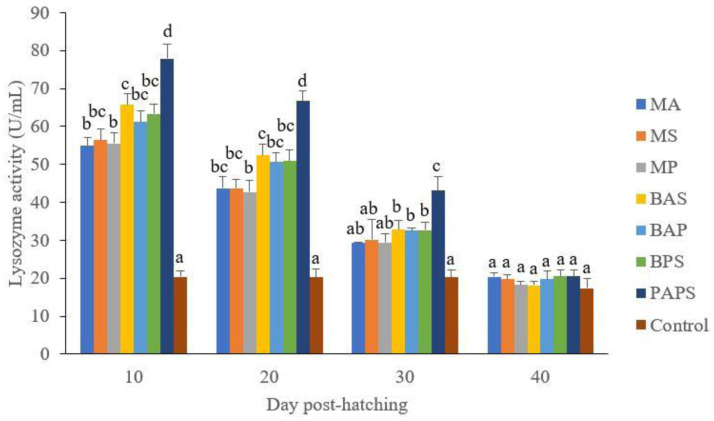
The lysozyme activity of tilapia offspring from maternal immunity produced by various types of vaccines at the ages of 10, 20, 30 and 40 days post-hatching (mean±SE). MA: *A. hydrophila* monovalent, MS: *S. agalactiae* monovalent, MP: *P. fluorescens* monovalent, BAS: *A. hydrophila* and *S. agalactiae* bivalent, BAP: *A. hydrophila* and *P. fluorescens* bivalent, BPS: *P. fluorescens* and *S. agalactiae* bivalent, and PAPS: *A. hydrophila, S. agalactiae,* and *P. fluorescens* polyvalent vaccines. Values with different superscripts a,b indicate that their corresponding means are significantly different (P<0.05) according to one-way ANOVA followed by Duncan’s test.

The lysozyme activity of broodstock vaccinated with PAPS (29.87–103.08 U/mL) was higher than other vaccines, and the lowest was in broodstock that was not vaccinated (27.65–33.89 U/mL) (P<0.05) ( Figure 3). Generally, the offspring from the broodstock vaccinated with PAPS had a higher lysozyme activity (77.81–43.11 U/mL) than those of other treatments (P<0.05) up to the 30 ^th^ day, the lowest was in the control (20.29–20.24 U/mL) The results showed that the application of PAPS in tilapia broodstock could increase lysozyme activity transferred to the offsprings ( Figure 4).

### RPS of offspring post-challenge

Offsprings that were 10, 20, 30, and 40 days old from the vaccinated broodstock had higher RPS than those from the unvaccinated broodstock after being challenged with bacteria. The offsprings from the broodstock that were vaccinated with PAPS had the highest RPS when challenged with 3 bacteria simultaneously (a combination between *A. hydrophila*, *S. agalactiae*, and *P. fluorescens*) ( Table 3) up to day 30. The RPS of the offspring vaccinated with PAPS were 86,11% (10 days old), 78,95% (20 days old) and 56,41% (30 days old). The immune response generated through maternal immunity only lasts up to 30 days and in the end, the immune response will be formed by the body of the offspring itself.

**Table 3.** The Relative Percentage Survival (RPS) of tilapia offspring from maternal immunity produced by various types of vaccines at the ages of 10, 20, 30 and 40 days post-hatching. The offspring were produced by broodstock vaccinated with various types of vaccines through intramuscular ( *i.m.*) injection (mean±SE).

Type of vaccine	Day post-hatching (day)			
	10	20	30	40
MA	66.67±4.81 ^a^	55.26±5.26 ^a^	41.03±2.56 ^a^	14.29±4.96 ^a^
MP	61.11±2.78 ^a^	50.00±6.96 ^a^	41.03±2.56 ^a^	14.29±4.96 ^a^
MS	63.89±2.78 ^a^	52.63±4.56 ^a^	43.59±2.56 ^a^	17.14±2.86 ^a^
BAP	72.22±2.78 ^a^	60.53±4.56 ^a^	46.15±4.44 ^ab^	11.43±7.56 ^a^
BAS	69.44±2.78 ^a^	60.53±4.56 ^a^	46.15±4.44 ^ab^	14.29±4.95 ^a^
BPS	69.44±7.35 ^a^	57.89±6.96 ^a^	43.59±2.56 ^a^	11.43±2.86 ^a^
PAPS	86.11±2.78 ^b^	78.95±2.63 ^b^	56.41±5.13 ^b^	20.00±2.86 ^a^

## Discussion

Efforts to produce seeds that are immune to several diseases were the best alternative to increasing Nile tilapia production. Furthermore, PAPSs for *A. hydrophila*, *S. agalactiae,* and *P. fluorescens* were able to improve the broodstock’s immune response which was then transferred to the offspring. This process was carried out in order to produce offspring that possess both lysozyme and antibodies and a high survival rate post-challenge test using pathogenic bacteria. This was better than the other treatments that made use of the bivalent and monovalent vaccines.

The results from the observation of the broodstock for 28 days showed that the total leukocyte ( Figure 1), phagocytic ( Figure 2), antibody titer ( Table 1), and lysozyme activity ( Figure 3), started to increase in week two post-vaccination. The broodstock vaccinated with PAPS showed a higher increase in the immune response compared to the others that were vaccinated with the bivalent, monovalent vaccines, and was the lowest in the unvaccinated broodstock ^28, 30, 33, 34, 51^. This showed that PAPS could increase the Nile tilapia broodstock’s immune response better than the other treatments.

The offspring produced from the broodstock that were vaccinated with PAPS had the highest antibodies ( Table 2) and lysozyme activity ( Figure 4) up to the 30 ^th^ day post-hatching period and was the lowest in the offsprings from the unvaccinated broodstock (P<0.05). This demonstrated that their strong immune response was transferred to their offsprings ^27– 29, 33, 34, 52^ through the egg yolk ^53^.

The results from the challenge test using pathogenic bacteria ( Table 3) showed that the offsprings that were produced using PAPS had a higher RPS compared to those from the offsprings produced from broodstocks that were treated using the monovalent and bivalent vaccines (P<0.05). This further showed that the vaccine treatment had adequately protected the fish from bacterial diseases with an RPS that was greater than 60% up to the 30 ^th^ day post-hatching period ^49^. RPS of the offspring vaccinated with formalin-inactivated vaccine in this study was higher at same time and lasted longer than the findings of Nurani *et al.* ^34^ on days 10 and 20, closely similar to the Sukenda *et al.* ^18^ and Pasaribu *et al.* ^54^, but higher on day 20. The high RPS in the offspring during the challenge test using pathogenic bacteria in PAPS treatment was due to the broodstock’s high number of leukocytes, phagocytic activity, the amount of antibody, and lysozyme activity transferred to the offsprings for protection against diseases. Meanwhile, in the control (unvaccinated), it only relies on immunity transferred naturally from the broodstock, whereas in the vaccinated broodstock, the offspring also get immunity from the broodstock which is induced by the vaccine. The existence of vaccine induction in the broodstocks can increase the total leukocytes, phagocytic activity, antibodies, and lysozyme activity of the offspring which are higher than the offspring produced from unvaccinated broodstocks. Thus, in the challenge test, the immune response of the vaccinated offspring is sufficient to control bacterial attacks, while the control offspring have not been able to control bacterial attacks. Compared to the Abu-elala *et al.* ^33^ study, the offspring immune response RPS was higher and could last up to 3 months, whereas in this study, the PAPS RPS vaccine was lower and only lasted up to days 30. Indicating that the role of the maternal immunity-derived polyvalent vaccine can protect the offspring from disease attack up to 30 days of age thus after 40 days of age the seeds only rely on the immune response naturally transferred from the mother or begin to produce their own immune response. The low RPS of the PAPS vaccine therefore requires improvement in the application of the maternal immunity method, such as the use of adjuvants, the use of quality tilapia broodstock, proper nutrition in terms of quality and quantity, and the application of biosecurity in the hatchery ^33^.

The role of leukocytes which consist of neutrophils, lymphocytes, and monocytes, is to infiltrate the infected area for rapid protection ^55^, stimulating the production of antibodies through the recognition of foreign bodies, including vaccines and pathogens during the challenge test in this study. The phagocytic activity occurs during phagocytosis, which involves antibodies and complements during opsonization. Furthermore, the total leukocyte parameter increases in line with other immune responses, such as the antibacterial lysozyme, which triggers the complement system and phagocytic cells ^56– 58^. It encourages phagocytosis by activating leukocytes and polymorphonuclear macrophages or through opsonization ^59^. The high number of leukocytes and a large amount of lysozyme in the treatment using PAPS which is similar to an infection by a pathogen indicated the success of PAPS in triggering the fish’s immune system when developing an immune response.

The offsprings produced by the broodstock that were vaccinated with PAPS were protected from infections by *A. hydrophila*, *S. agalactiae,* and *P. fluorescens*. However, the monovalent vaccines only protected the offsprings from one type of bacteria. This is one of the advantages of applying PAPS. The results of this study revealed that the application of PAPS produced broodstock and offspring with better immune responses than the bivalent and monovalent vaccines. Therefore, the development of a polyvalent vaccine is more prudent than that of bivalent or monovalent because of its ability to target more than one species of bacteria ^31, 51, 52, 60– 63^. The use of this type of vaccine caused the fish to respond to multiple antigens and form an immune response, thereby making it a strategic method in controlling bacterial diseases commonly found in culture and breeding environments ^33, 34, 52, 64^. Additionally, the application of polyvalent vaccines is more practical than the monovalent containing only one type of antigen. This showed that PAPS provided the most effective protection against diseases caused by pathogenic bacteria that often affect fishes, and thus is an ideal candidate for developing a polyvalent vaccine against bacterial infection.

## Conclusion

The results show that the application of the polyvalent vaccine against *A. hydrophila, S. agalactiae,* and *P. fluorescens* increased the antibody, lysozyme, total leukocytes, and phagocytic activity in Nile tilapia broodstock which was transferred to their offsprings, leading to a high RPS during the challenge test. Polyvalent vaccines derived from maternal immunity can protect offspring from disease up to 30 days of age. Therefore, it is possible to produce seeds of Nile tilapia that are immune to diseases caused by *A. hydrophila, S. agalactiae,* and *P. fluorescens*. This process could be carried out through the vaccination of the broodstocks using a polyvalent vaccine against *A. hydrophila, S. agalactiae,* and *P. fluorescens*.

## Ethical statement

Research using fish in Indonesia has not been regulated and therefore it does not require animal ethics. However, this research has received approval from the Ministry of Education and Culture of the Republic of Indonesia (No.: 004/PL.22.7.1/SP-PG/2019). In addition, this study applies the principle of the International Animal Welfare standards including the assurance of fish welfare during maintenance and the use of drugs during sampling.

## Data Availability

OSF: Underlying data for ‘ **Transfer of maternal immunity using a polyvalent vaccine and offspring protection in Nile tilapia, *Oreochromis niloticus*’**. https://doi.org/10.31219/osf.io/cnqdg ^65^ The project contains the following underlying data: Data on broodstock immune response, offspring immune response, and offspring RPS in tilapia, *O. niloticus* can be accessed on OSF Data are available under the terms of the Creative Commons Zero "No rights reserved" data waiver (CC0 1.0 Public domain dedication).
