# Supplementary material for: Transfer of maternal immunity using a polyvalent vaccine and offspring protection in Nile tilapia, Oreochromis niloticus
Source: F1000Res. 2023 Nov 10;10:966. Originally published 2021 Sep 24. [Version 4] doi: 10.12688/f1000research.52932.4 (PMC10521061; doi:10.12688/f1000research.52932.4)
Supplement: Peer review history

# Reviewer report: Reviewer response for version 4

**DOI:** 10.5256/f1000research.156437.r222370

**Published:** 2023-11-27

**Authors:** Indriyani Nur

**Affiliations:** 1 Department of Aquaculture, Faculty of Fisheries and Marine Science, Halu Oleo University, Kendari, South East Sulawesi, Indonesia

There will be no corrections from me to this manuscript any more. Everything has been revised by the authors. Therefore, it deserves to be approved.

Is the work clearly and accurately presented and does it cite the current literature?

Yes

If applicable, is the statistical analysis and its interpretation appropriate?

Yes

Are all the source data underlying the results available to ensure full reproducibility?

Yes

Is the study design appropriate and is the work technically sound?

Yes

Are the conclusions drawn adequately supported by the results?

Yes

Are sufficient details of methods and analysis provided to allow replication by others?

Yes

Reviewer Expertise:

Health of Aquaculture Organisms

I confirm that I have read this submission and believe that I have an appropriate level of expertise to confirm that it is of an acceptable scientific standard.

# Reviewer report: Reviewer response for version 3

**DOI:** 10.5256/f1000research.144612.r193310

**Published:** 2023-09-08

**Authors:** Indriyani Nur

**Affiliations:** 1 Department of Aquaculture, Faculty of Fisheries and Marine Science, Halu Oleo University, Kendari, South East Sulawesi, Indonesia

This study examines the presence of maternal immunity and the ability of polyvalent vaccines to protect offspring for several days. Some notes and corrections to the manuscript, including:

1. Please, describe how to collect eggs after spawning and hatching the eggs?
2. Please, describe in more detail the agglutination test, such as: (1) the serum dilution in each well, (2) how about wells for positive and negative control?
3. Make sure the information/symbol in the legend is the same as what is written in the caption figure or table.
4. What are the unit for agglutination titer in Tables 1 and 2?
5. On the 40th day, the observed parameters in all treatments were not significant, what does that mean on offspring?
6. There are several sentences that need to be revised according to the comments on manuscript. 

Is the work clearly and accurately presented and does it cite the current literature?

Yes

If applicable, is the statistical analysis and its interpretation appropriate?

Yes

Are all the source data underlying the results available to ensure full reproducibility?

Yes

Is the study design appropriate and is the work technically sound?

Yes

Are the conclusions drawn adequately supported by the results?

Yes

Are sufficient details of methods and analysis provided to allow replication by others?

Yes

Reviewer Expertise:

Health of Aquaculture Organisms

I confirm that I have read this submission and believe that I have an appropriate level of expertise to confirm that it is of an acceptable scientific standard, however I have significant reservations, as outlined above.

# Reviewer report: Reviewer response for version 2

**DOI:** 10.5256/f1000research.128337.r140937

**Published:** 2022-08-04

**Authors:** Najiah Musa

**Affiliations:** 1 Faculty of Fisheries and Food Science, Universiti Malaysia Terengganu, Kuala Nerus, Malaysia

Please refer to the highlights in the attached pdf.

 P3: Intro

 Para 1

 Streptococcus agalactia, Aeromonas hydrophila, Edwardsiella ictaluri, Flavobacterium columnaris, and Pseudomonas fluorescens are the pathogens. Disease refer to the infection, for examples Streptococcus agalactiae infection, Aeromonas hydrophila infection. Please revise. Consider 'Streptococcus agalactiae, Aeromonas hydrophila, Edwardsiella ictaluri, Flavobacterium columnaris, and Pseudomonas fluorescens infections'.

 Tilapia lake virus is the pathogen, the disease is tilapia lake virus disease. 

 Para 2

 'The offspring of fish' instead of 'their offspring' because 'they' refers to 'vaccines'

 Para 4

 It isn't applying polyvalent vaccines in offspring through maternal immunity, but 'application of polyvalent vaccines to confer maternal immunity in offspring'. Please rephrase.

 Methods

 Para 1, experimental animal

 What size was the spawning ponds that you managed to replace 25% of water daily? Please provide the dimension (LxWxD).

 Para 2, vaccine production

 The test vaccine. Please provide reasons why S.agalactiae and P. fluorescens were inactivated with 1% formalin while A.hydrophila inactivated with 0.6%? 

 P4: Para 1, vaccine treatments and administration

 Did you vaccinate both female and male broodstocks, or only female? Please mention.

 Revise 'However, before being injected with the vaccines, they were first anesthetized using MS-222, Sigma.'

 Consider: The fish were anesthetized using MS-222 (Sigma) prior to injection.

 Para 2

 25x30x1.2 m (L×W×D)

 Para 3

 Broodstock and larvae immune response.

 Intramuscular blood samples? Not caudal vein? 

 Para 4 

 Instead of 'post-spawning period', 'post-hatching period' will better reflect the offspring size. Please revise.

 How many offspring was ground in a tube, and what kind of tube was it?

 Why was there a range of 5-10 minutes centrifugation time? If the samples were centrifuged for different lengths of time, would it have affected the parameters later? Please clarify.

 Inactivate the complements or components? 

 What type of antigen? Whole-cell antigen?

 Para 5: Challenge procedures

 How long was the immersion in water containing pathogenic bacteria?

 P4 Results

 Broodstock total leukocyte dan phagocytic activity post vaccination

 PAPs did not result in highest total leukocyte in different time frames. BAP was highest on day 14.

 P5, para 1: Broodstock and offspring agglutination titers

 Please provide explanation as to why the unvaccinated fish (control) also show agglutination titer (although lower).

 Para 4, RPS of offspring post-challenge

 86.11% (10 days old), 78.95% (20 days old) and 56.41% (30 days old)

 Please provide explanation as to why the offspring of PAPS vaccinated broodstock encountered drops of RPS from 10 days old to 30 days old. 

 P5, Discussion, para 1

 Was carried out in order to produce...

 P7, Para 1

 The statement 'in the control (unvaccinated), there was no transfer of immunity from the mother' is not generally true. If the unvaccinated broodstocks have acquired immunity from prior infections, the immunity will have been passed on to the offspring as maternal immunity. It is only true that the offspring of the unvaccinated broodstocks did not have the vaccine-induced maternal immunity.   

 The statement 'the offspring hasn’t been able to produce their own immune response, so the total leukocyte, phagocytic activity, antibody, and low lysozyme activity' is not true. The offspring of the unvaccinated broodstocks did show immune response to the bacterial challenge but at the lower level compared with vaccinated group.

Is the work clearly and accurately presented and does it cite the current literature?

Yes

If applicable, is the statistical analysis and its interpretation appropriate?

Yes

Are all the source data underlying the results available to ensure full reproducibility?

Yes

Is the study design appropriate and is the work technically sound?

Yes

Are the conclusions drawn adequately supported by the results?

Yes

Are sufficient details of methods and analysis provided to allow replication by others?

Partly

Reviewer Expertise:

Aquatic animal health, microbiology

I confirm that I have read this submission and believe that I have an appropriate level of expertise to confirm that it is of an acceptable scientific standard, however I have significant reservations, as outlined above.

# Reviewer report: Reviewer response for version 1

**DOI:** 10.5256/f1000research.56264.r101122

**Published:** 2022-01-28

**Authors:** Chanagun Chitmanat

**Affiliations:** 1 Faculty of Fisheries Technology and Aquatic Resources, Maejo University, Chiang Mai, Thailand

The work is clearly and accurately presented. It is interesting research and I hope they can further study for farm application. However, the other serious bacteria pathogen is missing. Please add more review about Flavobacterium columnare. In addition, the viral pathogen doesn't be mentioned. It seems survival rates were quite low after bacterial challenge. Please discuss about low survival and how to improve it.

 This work, of course, has academic merit. This study was well designed, the details of the methods are enough and they could be replicated, and the statistical analysis was appropriate. However, please discuss more about the negative control. No challenge test for control groups? All the source data underlying the results were available to ensure full reproducibility and the conclusions are drawn adequately and supported by the results. However, I just wonder about the TiLV problem? Do you plan to produce vaccines?

 In addition to the previous comments, enclosed is the manuscript with some additional comments. ​​​​​​

Is the work clearly and accurately presented and does it cite the current literature?

Yes

If applicable, is the statistical analysis and its interpretation appropriate?

Yes

Are all the source data underlying the results available to ensure full reproducibility?

Yes

Is the study design appropriate and is the work technically sound?

Yes

Are the conclusions drawn adequately supported by the results?

Yes

Are sufficient details of methods and analysis provided to allow replication by others?

Yes

Reviewer Expertise:

fish immunology, fish diseases, aquaculture, aquaculture extension

I confirm that I have read this submission and believe that I have an appropriate level of expertise to confirm that it is of an acceptable scientific standard.

# Reviewer report: Reviewer response for version 1

**DOI:** 10.5256/f1000research.56264.r98048

**Published:** 2021-12-01

**Authors:** Najiah Musa

**Affiliations:** 1 Faculty of Fisheries and Food Science, Universiti Malaysia Terengganu, Kuala Nerus, Malaysia

Summary

 The study examined the transfer of vaccine-induced maternal immunity in Nile tilapia, *Oreochromis niloticus* against *Aeromonas hydrophila*, *Streptococcus agalactiae* and *Pseudomonas fluorescens*. The protective effects of monovalent, bivalent and polyvalent vaccines were compared. The relative percentage survival in immersion challenges, agglutination titers and lysozyme activities indicated that the polyvalent vaccine induced significantly better immune response compared with the bivalent, monovalent and unvaccinated groups.

 Part of the introduction is rather brief. Suggestion for improvement as follows:

1. Provide more references on vaccination in tilapia. The following two contain some of the relevant information
  https://doi.org/10.1002/aah.10099
  https://doi.org/10.1016/j.fsi.2019.04.052
2. Until which stage of offspring is the immune system not ready for immune response? Juvenile? Please elaborate more.
3. What types of Ig are transferable through eggs? Please elaborate. 

Part of the method description is rather brief and lacks references. Suggestion for improvements as follows:

1. Provide the reference for the two formalin concentrations used for inactivation of bacteria.
2. Mention the site of IM injection and provide the reference.
3. Mention the final bacterial concentration (cfu/mL) in the vaccines used at 0.4 mL/ kg.
4. Mention the size of the dirt ponds.
5. Detail the antigen preparation for direct agglutination test. Was it monovalent, bivalent or polyvalent?

Please see some additional annotations here.

Is the work clearly and accurately presented and does it cite the current literature?

Yes

If applicable, is the statistical analysis and its interpretation appropriate?

Yes

Are all the source data underlying the results available to ensure full reproducibility?

Yes

Is the study design appropriate and is the work technically sound?

Yes

Are the conclusions drawn adequately supported by the results?

Yes

Are sufficient details of methods and analysis provided to allow replication by others?

Partly

Reviewer Expertise:

Aquatic animal health, microbiology

I confirm that I have read this submission and believe that I have an appropriate level of expertise to confirm that it is of an acceptable scientific standard, however I have significant reservations, as outlined above.

## References

[1] : Vaccination of Tilapia against Motile Aeromonas Septicemia: A Review. *J Aquat Anim Health* .32(2) : 10.1002/aah.10099 65-76 10.1002/aah.10099 32331001

[2] : Efficacy of live attenuated vaccine derived from the Streptococcus agalactiae on the immune responses of Oreochromis niloticus. *Fish Shellfish Immunol* .2019;90: 10.1016/j.fsi.2019.04.052 235-243 10.1016/j.fsi.2019.04.052 31009810
